# Dataset on impact strength, flammability test and water absorption test for innovative polymer-quarry dust composite

**DOI:** 10.1016/j.dib.2020.105384

**Published:** 2020-03-05

**Authors:** Harrison Shagwira, Fredrick Mwema, Thomas Mbuya, Adeolu Adediran

**Affiliations:** a Materials, Design & Manufacturing Group (MADEM), Department of Mechanical Engineering, Dedan Kimathi University of Technology, Nyeri, Kenya; b Department of Mechanical & Manufacturing Engineering, University of Nairobi, Nairobi, Kenya; c Department of Mechanical Engineering, Landmark University, Kwara State, Nigeria

**Keywords:** Plastic, Quarry dust, Composite, Charpy impact test, Flammability test, Water absorption test, Construction

## Abstract

In this article data on impact strength, flammability and water absorption tests for innovative plastic-quarry dust composite is presented. The composites were prepared through moulding with virgin HDPE and PP plastics and quarry dust. The quarry dust was used at 0%, 5%, 20%, 40%, 60% and 80% weight percentages. The prepared samples were characterised for strength, fire resistance and hydrophobic properties using Charpy impact, flammability and water absorption tests respectively. For impact strength test was conducted according to ISO 179–1:2010 (E). The flammability test was conducted according to ASTMD 2863 while the water absorption test was carried out in accordance to ASTM D 570–98. These data illustrate the potential of the plastic quarry dust composite application in construction industry and model for regeneration of waste plastics for green building technologies.

**Table:** **Specification Table**

Subject	Material Science and Engineering
Specific subject area	Polymer Science
Type of data	Graphs, Image
How data were acquired	Charpy impact test was conducted using a Charpy impact tester (Zwick PSW 4J, Germany). Flammability test was carried out using Dynisco Limiting Oxygen Index Chamber (Model LOI 14273, USA). Water absorption test data was acquired by measuring the weights of the samples immersed in water using a digital weighing scale with an accuracy of 0.0001.
Data format	Raw and analysed.
Parameters for data collection	The Charpy impact test was conducted at room temperature (20 °C) with a 4-joule hammer used. The flammability was conducted by varying the concentration of Oxygen and Nitrogen in the burning chamber. The water absorption test was carried out at room temperature (20 °C) by varying the time the composite samples were immersed in water. The tests were done at atmospheric pressure and 6 bar pressure.
Description of data collection	The impact strength value on the scale was recorded after the failure of the sample after the impact. Samples were burnt vertically in the burning chamber for 3 minutes, reducing the amount of oxygen supply until the flame went off to obtain the flammability data. Samples were weighed before and after immersion in water for absorption test data.
Data source location	Institutions: TH Wildau/Chaka region/Dedan Kimathi University City/Town/Region: Wildau/Brandenburg & Nyeri Country: Germany/ Kenya/ Kenya Latitude and longitude (and GPS coordinates) for collected quarry dust samples/data: Chaka region (−0.337083, 36.997401)
Data accessibility	The analysed data is with this article and the raw data is provided as a supplementary file.

## Value of the data

• The data shows the mechanical strength, the fire behaviour and the water resistance characteristics of the plastic-quarry dust composites. The data is useful to evaluate the application of the prepared samples in the construction industry and model for plastic waste recycling in the construction industry.
• This data can benefit researchers of materials in construction industry, construction engineers and environmentalists in the quarry industries.
• The data can further be developed to evaluate specific applications in the building sector such as in roofing tiles, walling materials, etc.
• The data shows the influence of the appropriate choice of plastic-quarry dust proportions for optimal performance. It is an indicator of the need for future optimisation studies on the mixture constituents for enhanced performance of plastic-based composites as a construction material.

## 1 Data description

**Fig. 1:**
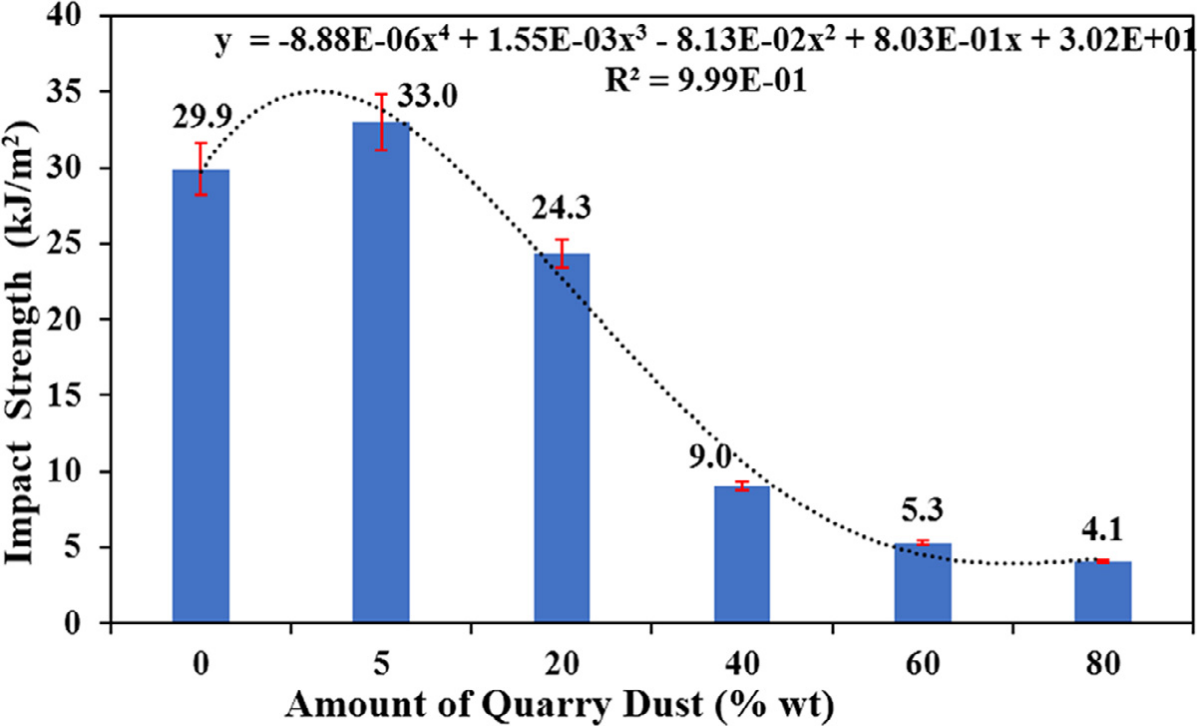
Charpy impact test plotted data of HDPE plastic-quarry dust for samples prepared with 0%, 5%, 20%, 40%, 60% and 80% weight concentrations of quarry dust.

**Fig. 2:**
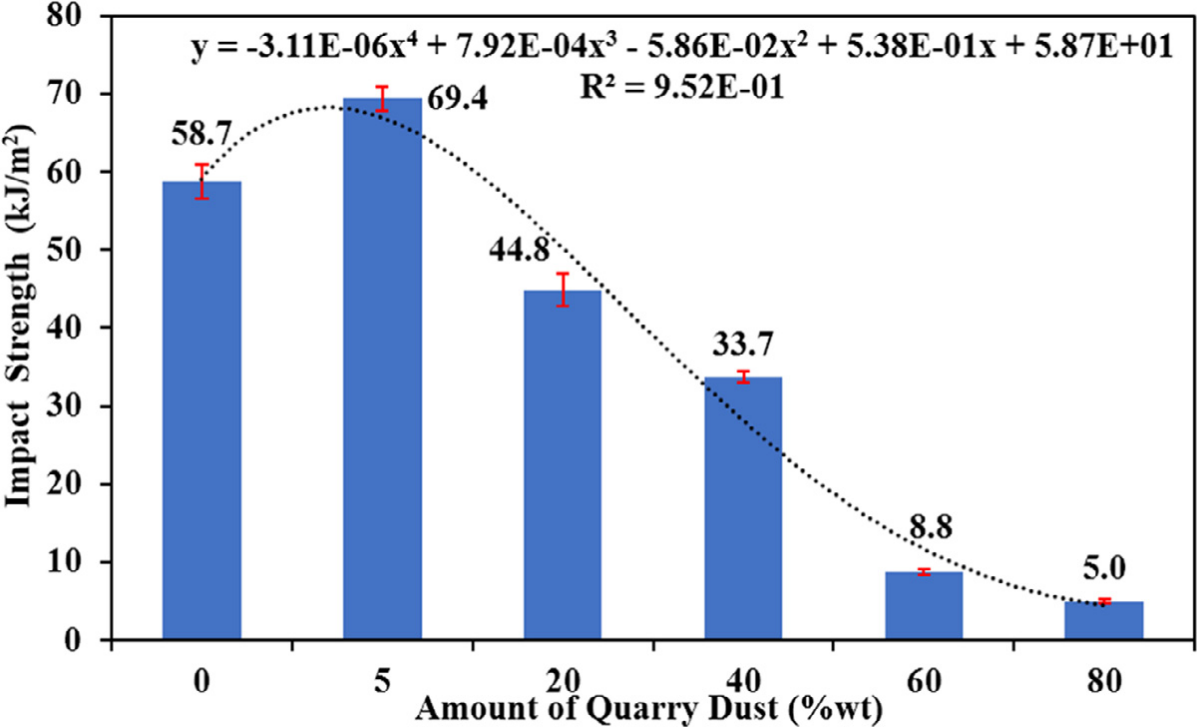
Charpy impact test plotted data of PP plastic-quarry dust for samples prepared with 0%, 5%, 20%, 40%, 60% and 80% weight concentrations of quarry dust.

**Fig. 3:**
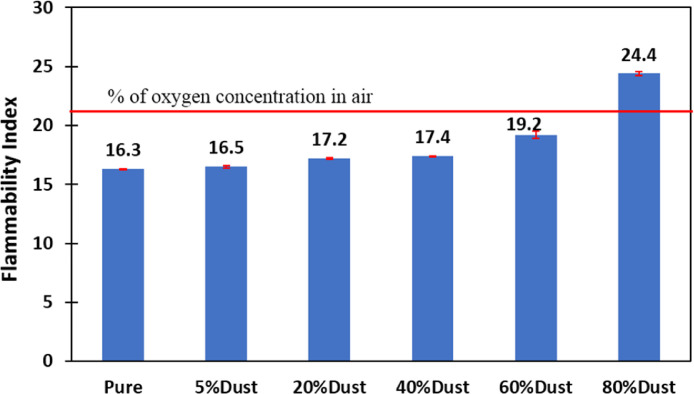
Flammability test plotted data of HDPE plastic-quarry dust for samples prepared with 0%, 5%, 20%, 40%, 60% and 80% weight concentrations quarry dust.

**Fig. 4:**
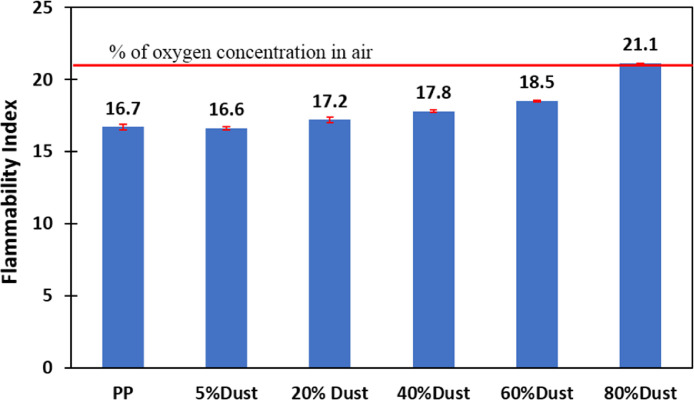
Flammability test plotted data of PP plastic-quarry dust for samples prepared with 0%, 5%, 20%, 40%, 60% and 80% weight concentrations quarry dust.

**Fig. 5:**
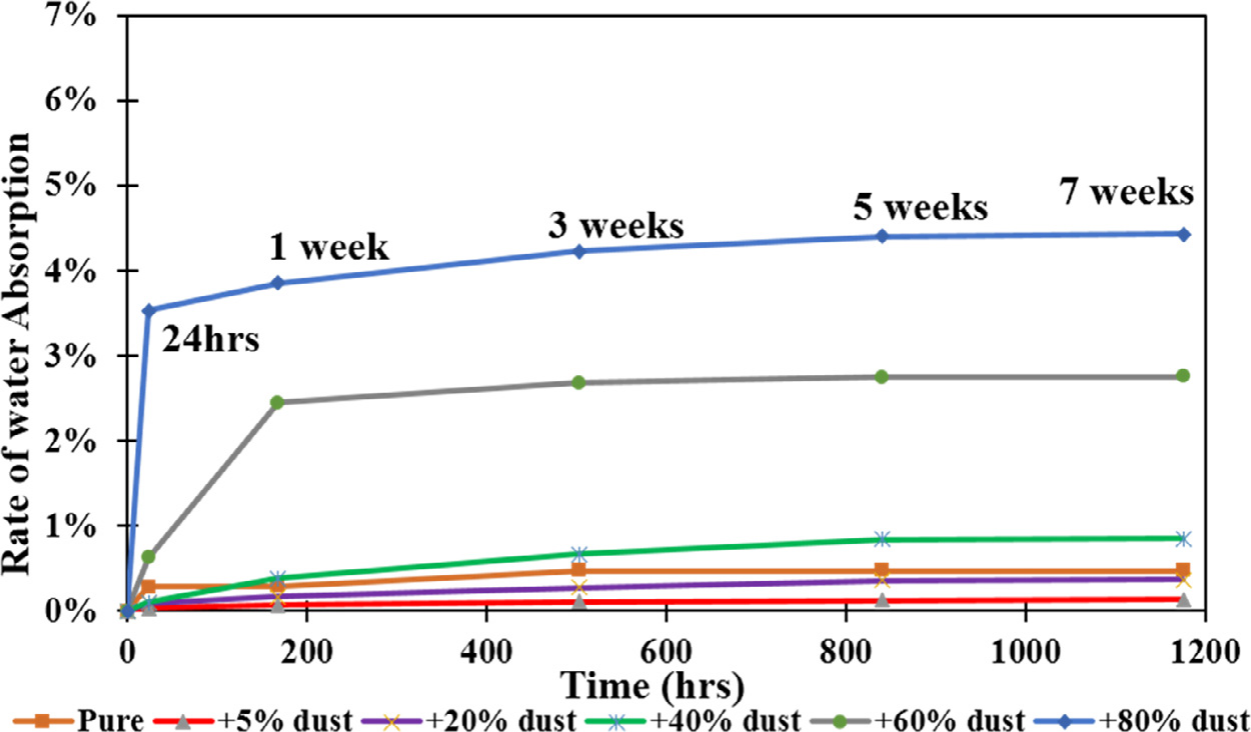
Water absorption test graph for HDPE-quarry dust-based composites Material at atmospheric pressure.

**Fig. 6:**
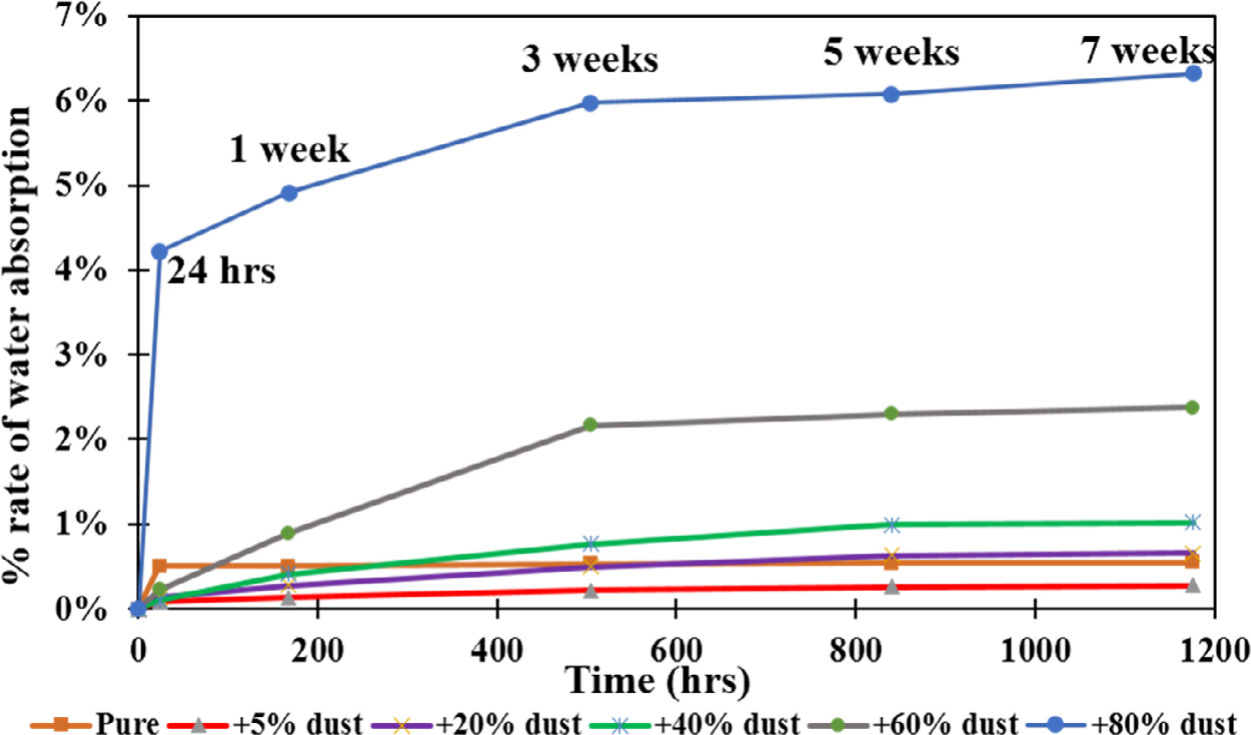
Water absorption test graph for PP-quarry dust-based composites Material at atmospheric pressure.

**Fig. 7:**
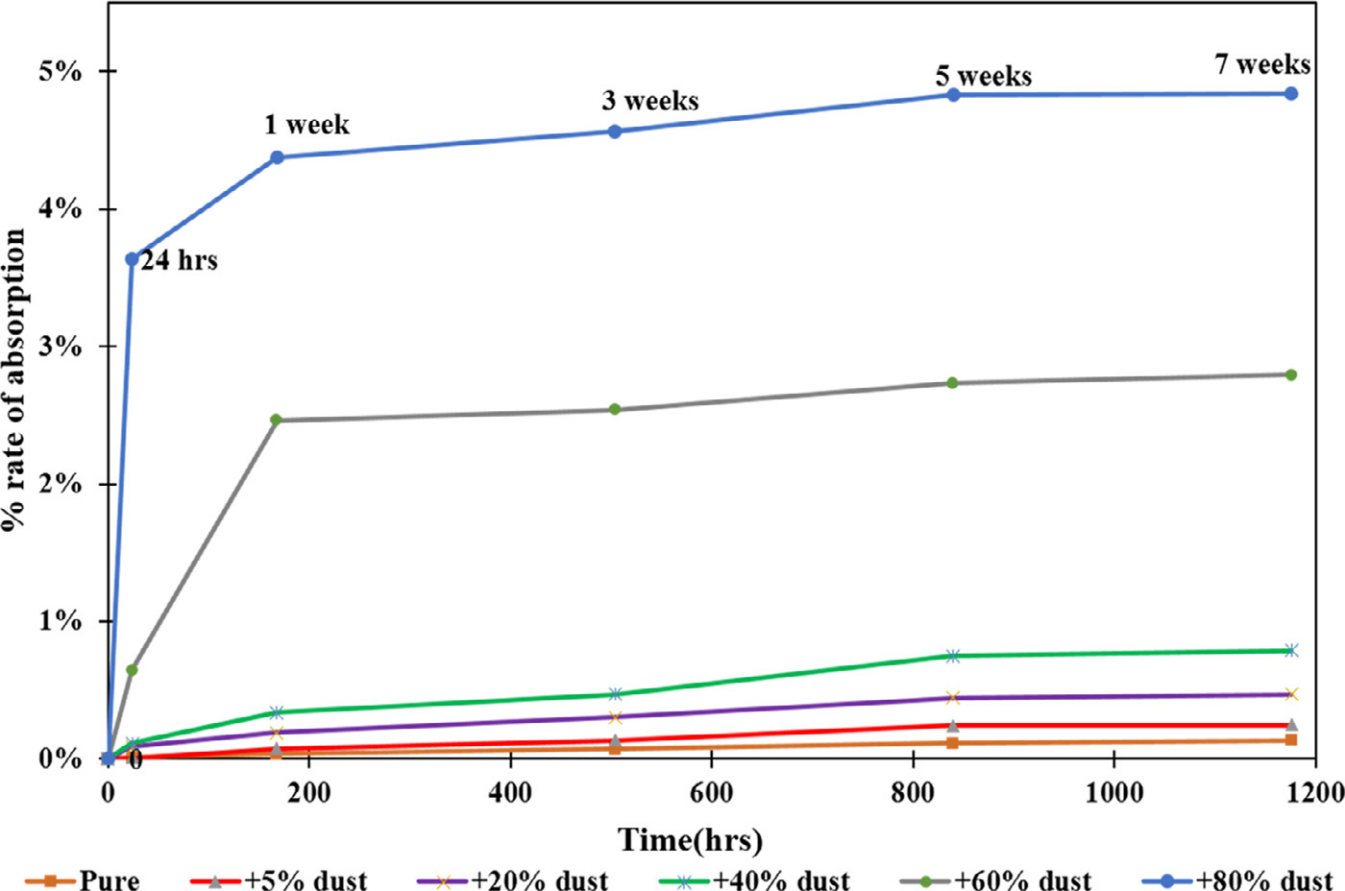
Water absorption test data for HDPE-quarry dust-based composites Material at pressure of 6 bars.

**Fig. 8:**
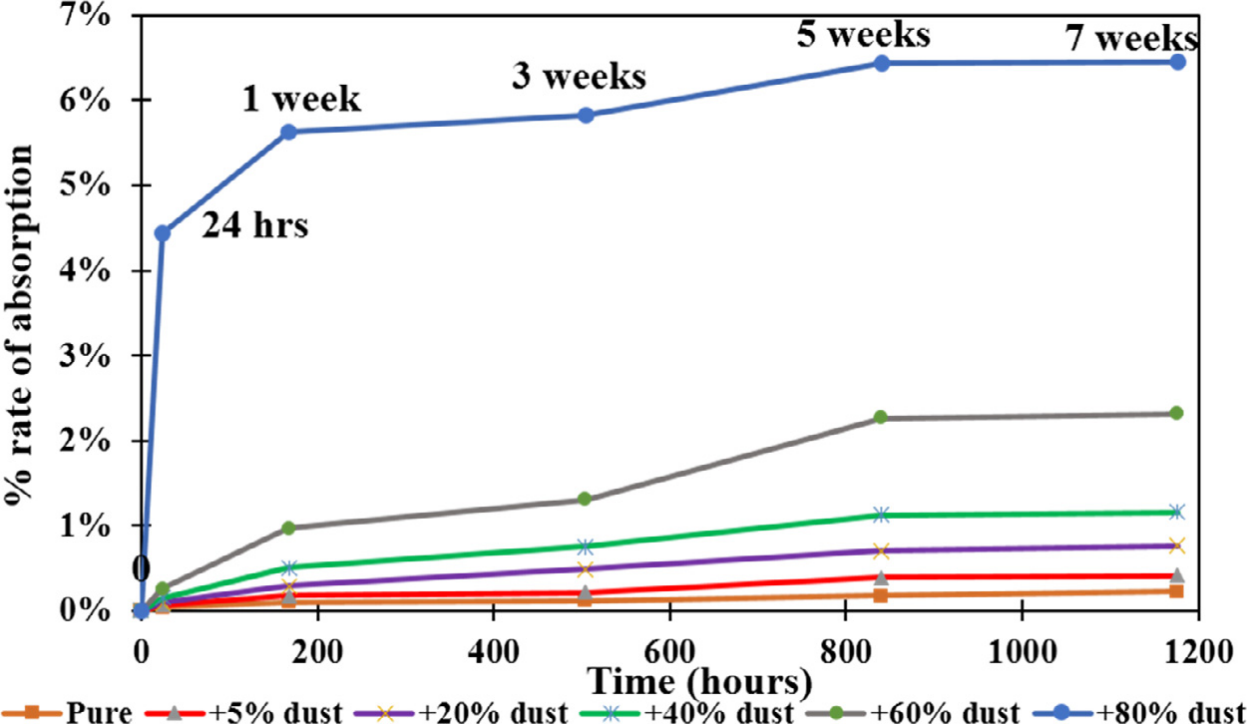
Water absorption test data for PP-quarry dust-based composites Material at pressure of 6 bars.

**Fig. 9:**
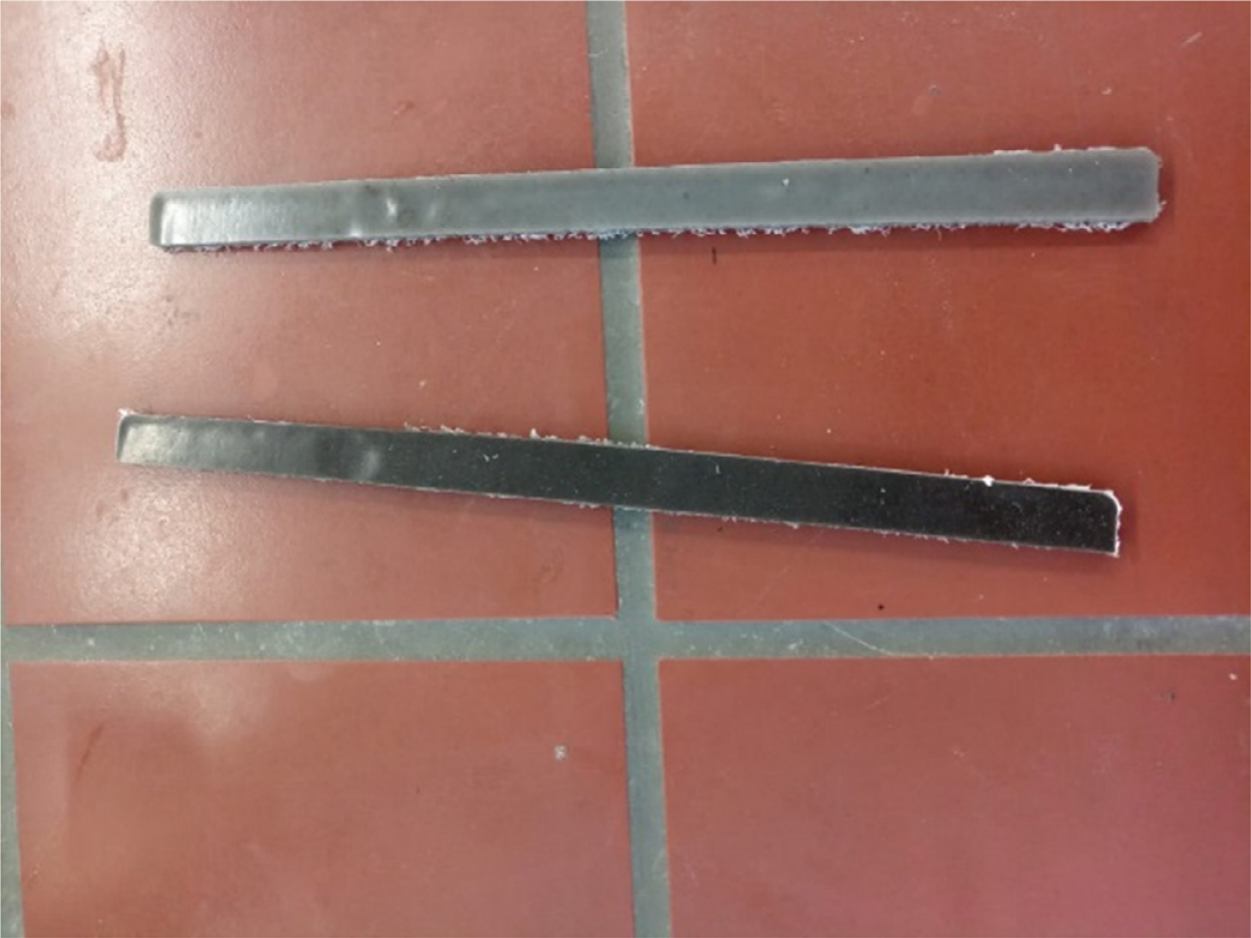
Plastic-quarry dust samples prepared by compression moulding and cut into 100 × 80 × 4 mm sizes according to ISO 179.

**Fig. 10:**
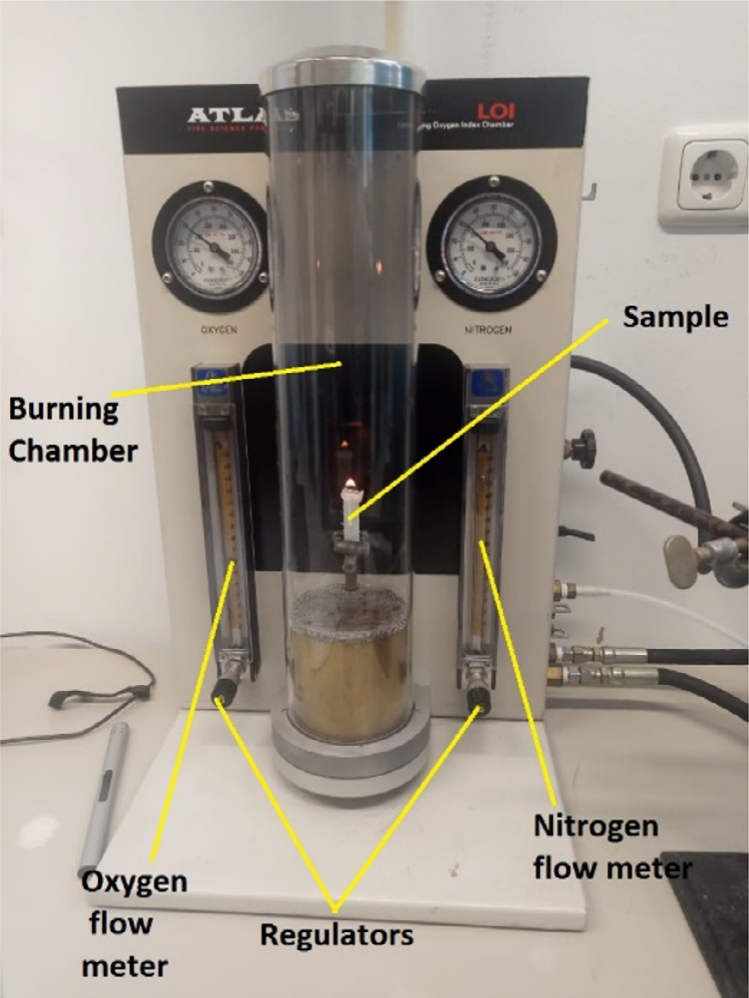
Pure PP burning inside the Dynisco Limiting Oxygen Index Chamber (Model LOI 14,273, USA).

**Fig. 11:**
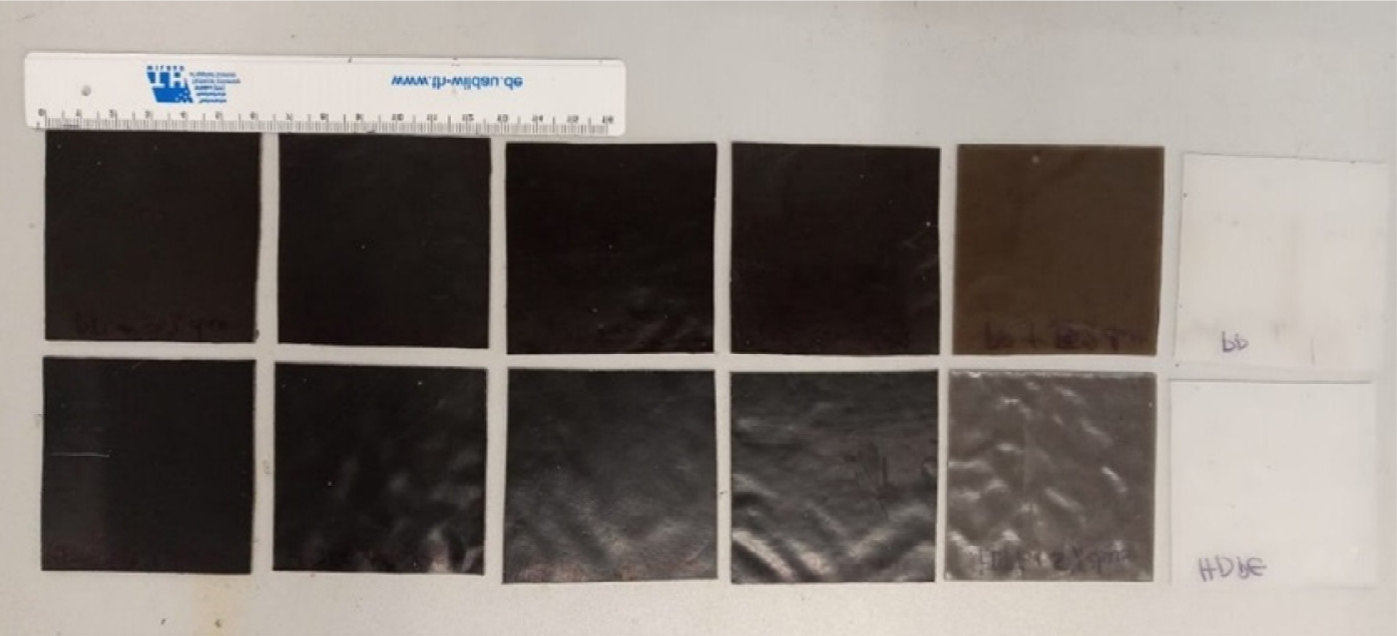
PP-quarry dust and HDPE-quarry dust samples prepared in 0%, 5%, 20%, 40%, 60% and 80% weight concentrations of quarry dust.

Fig. 1 shows the data for Charpy impact strength for HDPE-quarry dust composite prepared in 0%, 5%, 20%, 40%, 60% and 80% weight concentrations of quarry dust. Fig. 2 shows the data for Charpy impact strength for PP-quarry dust composite prepared in 0%, 5%, 20%, 40%, 60% and 80% weight concentrations of quarry dust. Additionally, Fig. 3 shows the limited oxygen indexes data of the HDPE composite samples having 0%, 5%, 20%, 40%, 60% and 80% weight concentrations of quarry dust. Fig. 4 shows the limited oxygen indexes data of the PP composite samples having 0%, 5%, 20%, 40%, 60% and 80% weight concentrations of quarry dust. The water absorption test for HDPE-quarry dust samples containing 0%, 5%, 20%, 40%, 60% and 80% quarry dust immersed in water under atmospheric pressure are shown in Fig. 5. Similarly, PP-quarry dust composite samples prepared under the same quarry dust composition and submerged in water under atmospheric pressure are shown in Fig. 6. HDPE-quarry dust and PP-quarry dust composite samples prepared in 0%, 5%, 20%, 40%, 60% and 80% weight concentrations of quarry dust exposed under pressure of 6 bar are displayed (Figs. 7 and 8). Fig. 9 shows the picture of the standard Charpy impact test samples, Fig. 10 shows the set up for limiting oxygen index test and Fig. 11 shows the pictures for the water absorption test samples.

## 2 Experimental design, materials, and methods

### 2.1 Charpy impact test

Charpy impact test was conducted according to ISO 179–1:2010 (E) using a Charpy impact tester (Zwick PSW 4 J, Germany). The samples were cut according to ISO 2818 into 100 mm × 10 mm × 4 mm sizes (sample shown in Fig. 1) [1]. After obtaining the energy reading from the Charpy test scale, the unnotched samples' Charpy impact strength a_CU_ (kJ/m^2^), were computed for each sample using Eq. (1). For each sample, ten (10) tests were conducted for statistical accuracy.

(1) $$a_{CU}=\frac{E_{C}\times 10^{3}}{h\times b}$$

Where:

E_c_ = the corrected stored energy in Joules,
h = the thickness of the test specimen (mm)
b = the width of the tests specimen (mm)

### 2.2 Flammability test

Flammability test was conducted according to ASTMD 2863 to accurately determine the relative flammability of the composites using the Dynisco Limiting Oxygen Index Chamber (Model LOI 14,273, USA). The samples were burnt inside the chamber (as shown in Fig. 2) while precisely regulating the amount of oxygen and nitrogen inside the chamber [1]. The test was conducted at 21 °C with three sets of gases velocity used, Norm Velocity (4.0 cm^3^/s), Low Velocity (3.2 cm^3^/s) and High Velocity (4.8 cm^3^/s). The readings of the flow metre were then recorded and used to calculate the oxygen index according to the following equation.

(2a) $$LOI=\frac{100\times O_{2}\;flow\;rate\left[\frac{cm^{3}}{\text{min}}\right]}{O_{2}\;flow\;rate\;\left[\frac{cm^{3}}{\text{min}}\right]+N_{2}\;flow\;rate\left[\frac{cm^{3}}{\text{min}}\right]}$$

### 2.3 Water absorption test

Water absorption test was conducted according to ASTM D 570–98 [1]. The composite's water absorption test was used for the determination of the quantity of water absorbed. The sizes of the samples were 60 mm × 60 mm × 1 mm as shown in Fig. 3. One set of samples were immersed in water under atmospheric pressure and another set of samples immersed in distilled water under 6 bars. An airtight stainless-steel container was used since it does not easily undergo corrosion. The specimens were immersed in distilled water at 23 °C for 24 h, 1 week, and after every two weeks until saturation was reached. Before weighing was done, the samples were wiped using a tissue to remove water on their surface. The absorption of water is expressed as the percentage increase in weight for each specimen as follows.

(2b) $$\%\;Increase\;in\;weight=\frac{wet\;weight,m_{2}-conditioned\;weight,m_{1}\;}{conditioned\;weight,m1}\times 100$$

## Appendix Supplementary materials

Image, application 1

Image, application 2

## References

[1] Chandramohan D., Presin Kumar A.J. (2017). Experimental data on the properties of natural fiber particle reinforced polymer composite material. Data Brief.

